# The spatial coexistence of TIGIT/CD155 defines poorer survival and resistance to adjuvant chemotherapy in pancreatic ductal adenocarcinoma

**DOI:** 10.7150/thno.86547

**Published:** 2023-08-18

**Authors:** Heng Ma, Xianlong Chen, Shengwei Mo, Xinxin Mao, Jingci Chen, Yilin Liu, Zhaohui Lu, Shuangni Yu, Jie Chen

**Affiliations:** Department of Pathology, Peking Union Medical College Hospital, Peking Union Medical College and Chinese Academy of Medical Science, Beijing, 100730, China.

**Keywords:** Pancreatic ductal adenocarcinoma, TIGIT/CD155, PD-L1, adjuvant chemotherapy, multiplex immunohistochemistry

## Abstract

**Background:** Targeting emerging T cell immunoreceptor with immunoglobulin and ITIM domain (TIGIT)/CD155 axis shows promise for restoring anti-tumor immunity, but its immune phenotypes and prognostic significance in a large cohort of pancreatic ductal adenocarcinoma (PDAC) are limited.

**Methods:** Three seven-color multispectral panels were rationally designed to investigate the protein expression, immune-microenvironmental feature, prognostic value, and the response to adjuvant chemotherapy of TIGIT/CD155 in 272 PDAC specimens using multiplex immunohistochemistry.

**Results:** We revealed low immunogenicity and high heterogeneity of the PDAC immune microenvironment featured by abundant CD3^+^ T cells and CD68^+^ macrophages and low infiltration of activated cytotoxic T lymphocytes. TIGIT and CD155 were highly expressed in PDAC tissues compared to paracancerous tissues. Tumor-infiltrating lymphocytes expressing TIGIT were correlated with high densities of CD45RO^+^ T cells; TIGTI^+^CD8^+^ T cells were associated with high infiltration of CD3^+^CD45RO^+^FOXP3^+^. CD155^+^CK^+^ were significantly related to high densities of CD3^+^ and CD3^+^CD8^+^CD45RO^+^ T cells. High positive rates for TIGIT in TCs, CD8^+^ T cells, and CD155 in macrophages were correlated with poor progression-free and disease-specific survival, respectively, and their clinical significance was correlated with PD-L1 status. Notably, spatial co-existence of TIGIT^+^CK^+^ or TIGIT^+^CD8^+^ and CD155^+^CD68^+^ indicated poor survival and resistance to adjuvant chemotherapy response in patients with PDAC.

**Conclusion:** Our findings suggest that targeting TIGIT/CD155 immunosuppressive axis may guide patient stratification and improve the clinical outcome of PDAC.

## Introduction

Pancreatic ductal adenocarcinoma (PDAC) is the most common aggressive pancreatic malignancy and remains a clinically lethal cancer with a 5-year relative survival rate of approximately 11% 1, 2. Current multiple-treatment strategies to slow or prevent PDAC exacerbation, include surgical resection, adjuvant chemotherapy (ACT), and immunotherapy. Regrettably, the overall response rate remains poor for resectable and unresectable patients 3. The underlying causes include delayed diagnosis, drug resistance, and immune resistance, and PDAC becomes highly refractory compared to any solid tumor type, which is a major medical challenge 4, 5. Therefore, advancing our understanding of PDAC biology to develop alternative multimodal therapeutic options is urgently required.

Tumor immune evasion driven by immune checkpoints creates a low-immunogenic ecosystem in multiple malignancies, which renders tumor-infiltrating lymphocytes (TILs) functionally inert and favors tumor progression. PDAC is among the most immune-resistant tumor types according to extensive profiling of the immune landscape 6, which is characterized by a barren tumor microenvironment (TME) featuring numerous immunosuppressive cell populations and is devoid of CD8^+^ T cells. The advent of targeted immune checkpoint inhibitors (ICIs) strategies has driven antitumor immunotherapy and may transform PDAC from immunologically “cold” to “hot”. However, one open-label, phase I clinical trial has demonstrated that a combination of nivolumab targeting programmed death-1 (PD-1) with standard chemotherapy (gemcitabine and nab-paclitaxel) has exhibited limited efficacy in patients with advanced PDAC 7. Similarly, the combination of dual immune checkpoint blockade against PD-L1 and cytotoxic T-lymphocyte antigen 4 (CTLA-4) in patients with metastatic PDAC has shown a disappointing overall response 8, suggesting the existence of unexploited checkpoints that hinder this promising therapeutic strategy.

T cell immunoreceptor with immunoglobulin and ITIM domain (TIGIT) (also known as VSIG9, Vstm3, and WUCAM) is a coinhibitory receptor belonging to the immunoglobulin superfamily 9 and has sparked enormous interest in relation to cancer immunity. TIGIT was initially identified and named by Grogan et al. through genomic searches for genes specifically expressed in activated human T cells, which play a significant role in modulating T cell-mediated innate and adaptive immunity 10, 11. TIGIT is mainly expressed in effector CD8^+^ T cells, regulatory CD4^+^ T cells, and natural killer cells. TIGIT co-expressed with programmed cell death protein 1 (PD-1) increases the expansion of tumor antigen-specific CD8^+^ T cells and CD8^+^ TILs in multiple preclinical models 12, 13. CD155 (PVR) is the main specific ligand for TIGIT and is mainly expressed by tumor cells (TCs), macrophages, and dendritic cells 11, 12. Engagement between TIGIT and CD155 initiates major inhibitory signaling cascades that trigger interleukin (IL)-10 secretion and decreased production of IL-12, leading to T cell function exhausted 14, 15. A phase 1 clinical study (NCT02964013) using anti-TIGIT antibody vibostolimab and pembrolizumab indicated that the objective response rate in patients with advanced solid tumors was 7%, showing tolerability and antitumor activity 16. Therefore, the TIGIT/CD155 axis can be further explored and rationally leveraged as an attractive target of immunotherapy for solid tumors.

PDAC shows inter-intra-tumor heterogeneity and high TME complexity of the TME; therefore, a comprehensive analysis of the spatial interplay between the immune cell (ICs) populations and TCs are warranted for rational tumor control. Unlike conventional immunohistochemistry (IHC), multiplex IHC (mIHC), as a multi-dimensional imaging technology, maps the spatial distribution of the ICs populations *in situ*, allows for simultaneous single-cell phenotyping of multiple immune markers while retaining their spatial information, and explores how immune subsets spatially and cooperatively contribute to tumor progression and patient outcomes 17-19. In this study, we rationally designed three multiplexed panels each with seven-marker labels based on mIHC and computational digital imaging technologies to characterize the TIGIT/CD155 axis-mediated immunosuppressive landscape and to investigate their correlations with multiple immune markers (CD8, CD3, forkhead box P3 (FOXP3), granzyme B (GB), CD45RO, PD-L1) and potential clinical and prognostic significance in archival PDAC specimens.

## Materials and Methods

### Patients and tumor tissue specimens

A total of 272 patients diagnosed with primary PDAC between January 2015 and July 2019 were consecutively recruited from the pathology archives of the Peking Union Medical College Hospital (PUMCH) (Beijing, China). Approval for this study was obtained from the Institutional Review Board of Peking Union Medical College Hospital (reference number S-K1593) and was performed following the standards of the Declaration of Helsinki; informed written consent was required from all enrolled participants or consent was waived due to the retrospective data being anonymized in some cases. Patients with neoadjuvant treatment, unclear prognostic information, and who died due to postoperative complications were excluded from the current study. Clinicopathological information of PDAC patients was retrospectively collected from medical records and pathology reports, and follow-up information was recorded by telephone interviews from hospitalization to endpoint emerged on October 10, 2020. Progression-free survival (PFS) was the span from the first surgery to tumor progression, distant relapse, or the last follow-up appointment. Disease-specific survival (DSS) was the time interval between the date of resection and the time of death caused by PDAC. In our study cohort, 199 of the 272 patients received adjuvant chemotherapy, of which 65 patients were treated with TS-1 (tegafur‒gimeracil‒potassium oxonate), 57 patients were treated with gemcitabine, 45 were treated with TS-1 combined with gemcitabine, 23 patients received 5-fluorouracil, while 9 patients were treated with gemcitabine and capecitabine.

To select the regions of interest for tissue microarray (TMA) construction, hematoxylin and eosin-stained tissue sections were strictly re-evaluated, and representative regions of interest were marked on the slides by two board-certified pathologists (J.C. and S.N.Y). Then, selected areas were comparatively punched from formalin-fixed and paraffin-embedded blocks using a Tissue Microarrayer (MiniCore, Mitogen, Hertford, UK) and re-embedded into recipient blocks. Then, TMA sections with a diameter of 2.0 mm were prepared for the following experiment.

### mIHC and multispectral imaging

mIHC staining was performed on TMA slides using an Opal Polaris^TM^ 7-color Kit (Akoya Biosciences, MA, USA) as previously described 20. Briefly, 4-µm-thick TMA sections were baked and melted at 70 °C for 40 min and then deparaffinized in xylene and rehydrated using graded ethanol, respectively. Microwave-mediated heat-induced antigen retrieval with AR6 buffer (pH, 6.0) was performed. Cover tissue sections were blocked with Antibody Diluent/Block for 10 min at 37 ℃ and incubated with the indicated primary antibodies for 2 h at 37 °C. The details about antibody concentration, staining condition, and Opal fluorophores for each immune marker are listed in **Table S1** and were optimized singly for good spectral unmixing. The unstained negative sections were applied to deduct the autofluorescence spectrum of the PDAC tissues. Then, the slides were serially incubated with Opal Polymer HRP Ms + Rb and subjected to tyramide signal amplification (TSA) with 300 μL Opal Woking Solution. After microwave treatment and antibody stripping, another round of staining can be performed for additional target detection. Until all targets were labeled, the cell nuclei were stained with DAPI and covered with a mounting medium.

The stained sections were simultaneously scanned by the Vectra multispectral slide scanner (Vectra 3.0, PerkinElmer). A3 × 3 (2793 × 2094 μm; 20 × field resolution) fixed stamps were used to capture each core to create a spectral library based on different fluorophores, which consisted of separate and composite multispectral images. Cell phenotypes including the features of fluorophore and nucleus were labeled by indicated immune markers. CK^+^ denotes PDAC cells, CD68^+^ denotes macrophages, and CD8^+^ denotes cytotoxic T lymphocyte cells. Then, all spectrally unmixed images (*n* = 3783) with annotation features were reevaluated and reclassified by two trained pathologists (H.M. and X.L.C.).

### Conventional IHC

IHC staining was performed as previously described 21. Briefly, whole tissue sections and TMAs sections were air dried, deparaffinization, and hydrated; then antigens were retrieved (citrate buffer [pH 6.0] or EDTA buffer [pH 9.0]), endogenous peroxidase quenched, and samples blocked in goat serum (ZLI-9096). Then whole sections were incubated with primary antibodies for mIHC at 4 ℃ overnight. Next, all slides were incubated with secondary antibodies and counterstained with Mayer's hematoxylin. All cover-slipped TMAs were automatically scanned at 400 × magnification using NanoZoomer S360 (Hamamatsu, Japan). The semi-quantitative immunoreactive scores for TIGIT, CD155, CD3, CD8, and FOXP3 were used to evaluate the immunostaining (H.M. and XL.C.), and any discrepancies were resolved by a consensus pancreatic cancer diagnostic pathologist (J.C.). Markers staining was quantified by counting the positive cells in the four random fields (at 400 × magnification), and the mean value was adopted for statistical analysis.

### TIGIT, CD155, and TILs evaluation

Given that peripheral red blood cells (RBCs) highly expressed TIGIT and CD155 in part of PDAC patient's tissues, which resulted in the incorrect recognition of positive ICs by Inform software after high-sensitivity mIHC staining. Thus, the expression of TIGIT, CD155, CD3, CD8, FOXP3, GB, CD45RO, PD-1, and PD-L1 on TILs or TCs was manually assessed and quantified by two investigators (H.M. and X.L.C.) who were blinded to any clinical information. Discrepant results were resolved by consulting a third expert gastroenteropancreatic pathologist (J.C.). TIGIT and CD155 expression was estimated in TCs and TILs separately. TCs (CK^+^) that were TIGIT- or CD155-positive with at least 1% cell staining in each case showed membranous staining and were defined as TIGIT^+^CK^+^ and CD155^+^CK^+^, separately. TILs are heterogeneous lymphocytes in the tumor stroma, including T cells and NK cells, CD3^+^ and CD8^+^ T cells account for the main components. TILs with ≥ 1% TIGIT-staining were defined as TIGIT^+^TILs. CD155-positive macrophages were scored if at least 1% of cells were co-expressed with CD68 and CD155, and defined as CD155^+^CD68^+^. The number of effects/memory cytotoxic T lymphocyte (CD3^+^CD8^+^CD45RO^+^), activated cytotoxic T lymphocytes (CD3^+^CD8^+^GB^+^), and memory/regulatory T lymphocyte (CD3^+^CD45RO^+^FOXP3^+^) was enumerated from each TMA core due to the small number of colocalizing positive cells. For scoring of CD3, CD8, FOXP3, and CD45RO, the number of positive cells was counted from five representative views in a high-power field (HPF, ×400 magnification), and the mean was used for statistical analysis. The best cut-off values for each marker were determined by X-tile (Yale University, USA) for comparative analysis.

### Western blotting

Western blotting was conducted as previously reported 22. Briefly, we consistently collected PDAC surgical resection pathological specimens without any bias or pre-screening during 2019, from which 42 paired PDAC tissues and paracancer tissues were randomly selected for western blot analysis. 100 mg samples were lysed in radioimmunoprecipitation assay (RIPA) buffer (APPLYGEN, C1053+) containing 1% protease inhibitor and 1% phosphatase inhibitors for 30min on ice. Then, centrifugation at 12,000 × g for 15 min at 4 °C, the supernatant proteins were quantified using BCA assay (Thermo Scientific, BCA protein assay kit, 23225). Thirty micrograms of protein were mixed with 5 × loading buffer and denatured in a 95 °C water bath. Primary antibodies against TIGIT rabbit mAb (Abcam, ab243903, 1:1000), CD155 rabbit mAb (Cell Signaling Technology, #81254, 1:1000), and anti-β-Actin (D6A8) rabbit mAb (Cell Signaling Technology, #8457, 1:1000) were incubated with PDAC tissues overnight at 4°C for the following blot detection.

### PD-L1 evaluation

PD-L1 expression is quantified by a composite positive score (CPS), which is calculated by dividing the number of PD-L1-stained cells (TCs, infiltrating lymphocytes, and macrophages) by the total number of TCs and multiplying by 100. Samples of CPS ≥ 1 are positive for PD-L1.

### Statistical analysis

All statistical analyses were analyzed by IBM SPSS version. 22 or GraphPad Prism version 9. For categorical variables, the χ^2^ test or Fisher's exact test was used to evaluate the relationship between TIGIT and CD155 and clinicopathological parameters. Mann-Whitney *U* test was used for the comparison of continuous variables. Spearman correlation analysis was used to assess all correlations between the ICs population. The Kaplan-Meier analyses with log-rank tests, univariate, and multi-variable-adjusted Cox proportional hazards regression analyses were used to evaluate the survival and determine the independent prognostic factors, respectively. P-value < 0.05 indicated statistical significance.

## Results

### Three multi-spectral panels characterize immune heterogeneity in the immune ecosystem of PDAC

**Figure 1:**
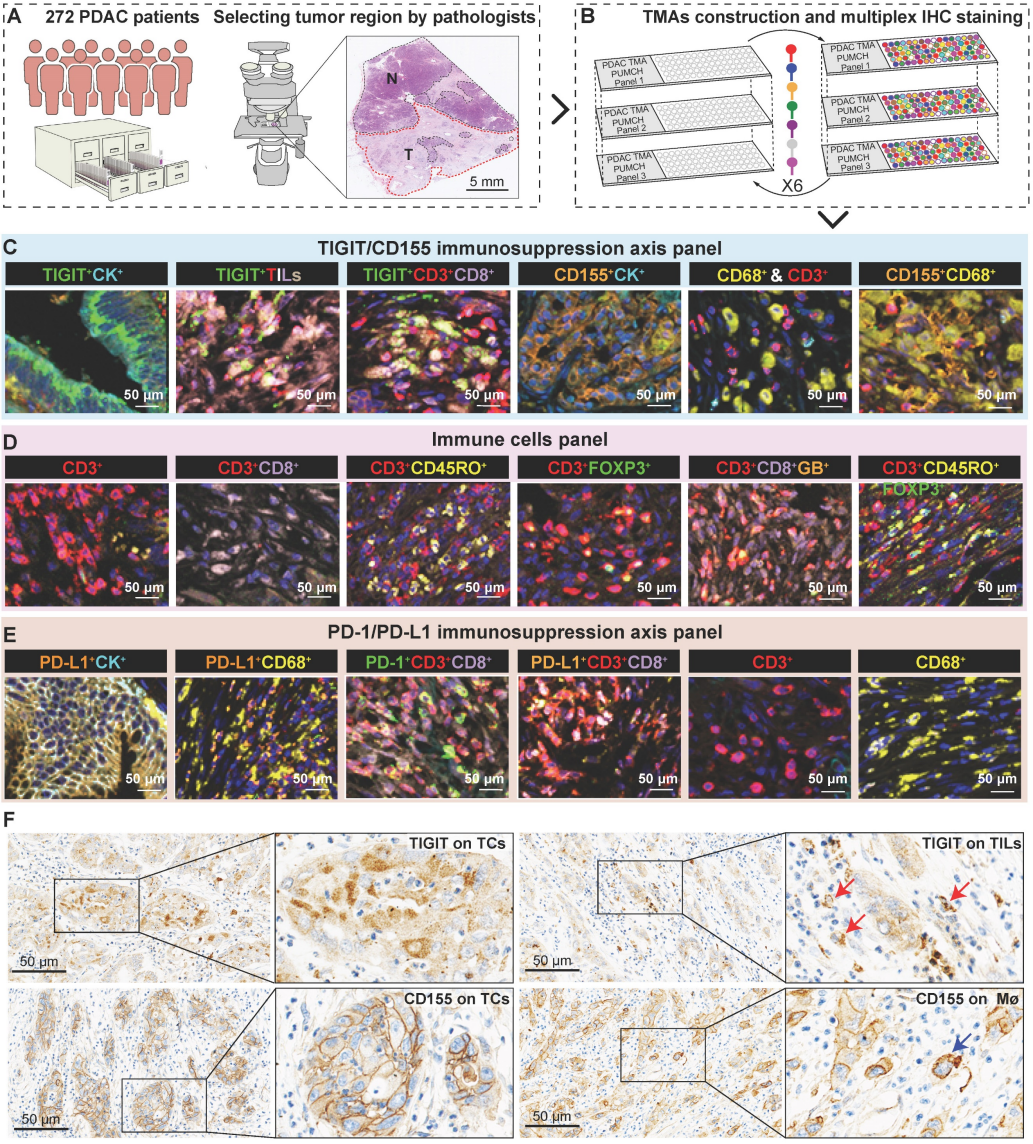
**Multiplex immunohistochemistry profiling of immune cells landscape in archived PDAC patient tissue samples. (A)** Schematic representation of the region of interest selection from hematoxylin and eosin-stained PDAC sections for tissue microarray (TMA) construction. **(B)** A flow diagram of multiplex IHC staining. **(C-E)** Representative colocalization immune markers-stained composite images from 272 PDAC TMA cores, including TIGIT/CD155 immunosuppression axis panel **(C)**, immune cells panel **(D)**, and PD-1/PD-L1immunosuppression axis panel **(E)**. Representative single-stained images were shown in the right panels of Supplementary Figure 2A**-**C. **(F)** Representative IHC images of TIGIT-positive on tumor cells (TCs) and CD8^+^ T cells and CD155-positive on TCs and macrophages in PDAC tissues. Scale bar = 50 μm. *P < 0.05 indicates statistically significant.

To fully investigate the immune landscape labeled by TIGIT/CD155, immune markers, and PD-1/PD-L1, we rationally designed three seven-color multispectral panels and each containing 272 archived PDAC patient specimens **(Figure 1A-B, Figure S1A-C)**. Positive TIGIT expression on TCs, TILs (mainly CD3^+^ T cells) was observed in 82.95% and 88.83% of the samples, respectively; Positive CD155 on TCs and CD68^+^ macrophages were observed in 79.09% and 61.83% of the samples, respectively** (Figure S1D)**. These positive staining cells were differentially distributed across distinct subregions in tumors and exhibited a quite different staining intensity **(Figure S1E).** Notably, according to co-expression and colocalization, we identified five heterogeneous phenotypes in TIGIT/CD155 immunosuppression axis panel, including TIGIT-expressing malignant cells (TIGIT^+^CK^+^), TILs expressing TIGIT (TIGIT^+^ TILs), cytotoxicity T lymphocytes expressing TIGIT (TIGIT^+^CD3^+^CD8^+^), TCs expressing CD155 (CD155^+^CK^+^), and macrophages expressing CD155 (CD155^+^CD68^+^). TIGIT and CD155 were widely expressed in TCs and ICs in PDAC and exhibited cytoplasmic/membranous staining **(Figure 1C, Figure S2A)**. In the immune cells panel, we identified cytotoxicity T lymphocytes (CTLs) (CD3^+^CD8^+^), memory T lymphocytes (CD3^+^CD45RO^+^), regulatory T lymphocytes (CD3^+^FOXP3^+^), activated CTLs (CD3^+^CD8^+^GB^+^), and memory/regulatory T lymphocytes (CD3^+^CD45RO^+^FOXP3^+^) were differentially distributed across distinct subregions in PDAC tissues **(Figure 1D, Figure S2B)**. In the PD-1/PD-L1 immunosuppression axis panel, PD-L1 was highly expressed on tumor cells and macrophages, while PD-1 was more co-expressed with ICs, PD-1^+^CD3^+^CD8^+^, and PD-L1^+^CD3^+^CD8^+^ ICs were less abundant in TILs (**Figure 1E, Figure S2C**). Additionally, TIGIT and CD155 were highly expressed in TCs, both exhibiting membranous/cytoplasmic staining in traditional IHC (**Figure 1F**). Interestingly, TIGIT and CD155 were highly co-expressed in tumor compartments but showed subtle differences, TIGIT was located in the intraluminal membranes, and CD155 was expressed in the extraluminal membranes of pancreatic duct cells (**Figure 1F**). We also observed TIGIT expression on TILs and CD155 expression in macrophages, both of which were topologically consistent with mIHC results (**Figure 1F**). Together, these heterogeneous distributions of immune variables associated with TIGIT and CD155 in tumor tissues highlight the spatial immune complexity in PDAC.

### Expression of TIGIT and CD155 and their correlation with clinicopathological features in PDAC

**Figure 2:**
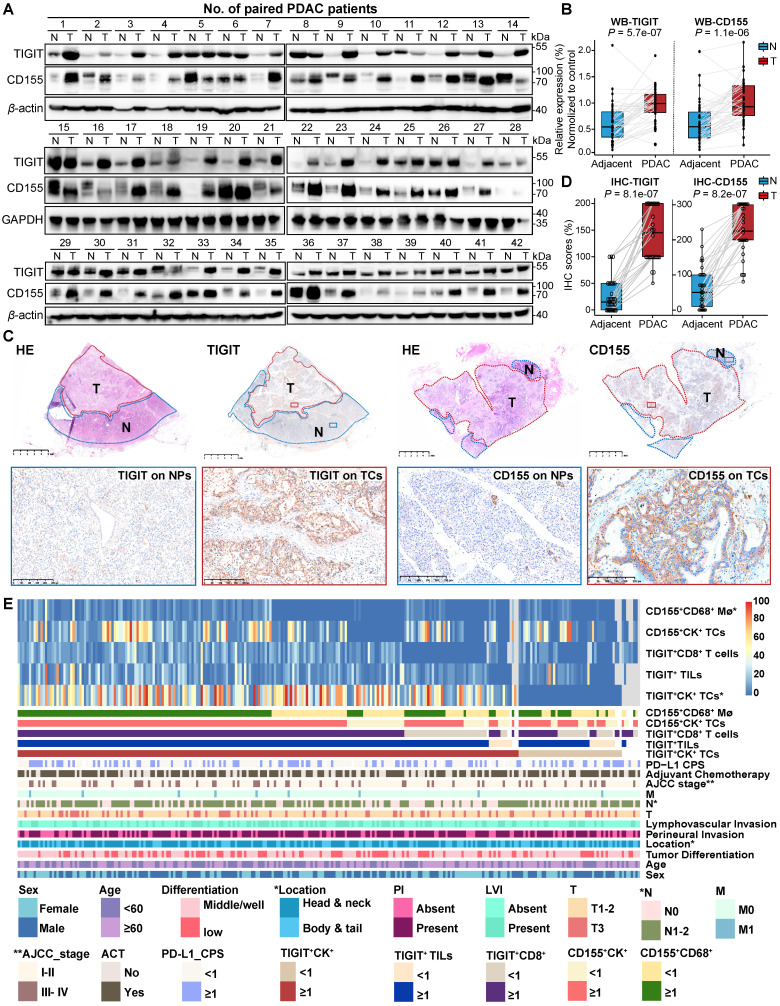
** Expression of TIGIT and CD155 and their correlation with clinicopathologic features in PDAC patient samples. (A-B)** Western blot analyzing the protein expression of TIGIT and CD155 in 42 paired PDAC and adjacent paracancerous tissues **(A)**, quantified by ImageJ **(B)**. Wilcoxon matched-pairs signed rank test. *P* < 0.05. **(C-D)** The representative IHC staining images of TIGIT and CD155 in 32 human PDAC whole tissue sections that contain tumor and paracancerous tissues **(C)**, were quantified by H-score **(D)**.** (E)** Heatmap displayed the proportions and overlap between TIGIT/CD155-positive phenotypes and clinicopathologic features. Mø, macrophages; TCs, tumor cells; TILs, tumor infiltration lymphocytes; CPS, combined positive scores; AC, adjuvant chemotherapy; PI, perineural invasion; LVI, and lymphovascular invasion. T, tumor stage; N, node stage; M, distant metastasis. *P < 0.01; **P <0.001.

To conduct a more comprehensive comparison of TIGIT and CD155 expression levels in tumor and adjacent non-malignant tissues, a total of 42 paired PDAC tissues and adjacent normal tissues were randomly selected and subjected to western blotting. TIGIT and CD155 were highly expressed in tumor tissues compared to those in adjacent normal tissues in a pairwise manner (**Figure 2A-B**). Analysis of mRNA expression of TIGIT and CD155 from The Cancer Genome Atlas (TCGA) showed similar results (**Figure S3A**). Notably, we further examined the expression of TIGIT and CD155 on the whole tissue section using immunohistochemical analysis from 32 PDAC samples which contained both tumor tissues and adjacent paracancerous tissues. We confirmed that TIGIT and CD155 were significantly highly expressed in tumor cells compared with adjacent normal pancreas cells (**Figure 2C-D**) (**Figure S3B-D**). Furthermore, we evaluated the correlation between the densities of immune checkpoints TIGIT/CD155-positive cells and distinct clinicopathological parameters. Unfortunately, no significant dependencies were noticed between TIGIT positivity on TCs, TILs, and CD8^+^ T cells with respect to clinicopathological factors, except for TIGIT positivity on TCs, which was related to a higher American Joint Committee on Cancer (AJCC) stage (III-IV) (*P* < 0.001) and was more frequently observed in tumors with higher node stage (N1-2) (*P* = 0.001) (**Figure 2E, Table S2**). Interestingly, CD155 positivity on CD68^+^ macrophages instead of that on TCs was associated with head or neck localization in the pancreas (*P* = 0.04) (**Figure 2E, Table S3**). Therefore, high expression of TIGIT and CD155 may modulate the functionality of different immune populations and favor PDAC progression.

### Prognostic values of the TIGIT/CD155 axis in patients with PDAC

**Figure 3:**
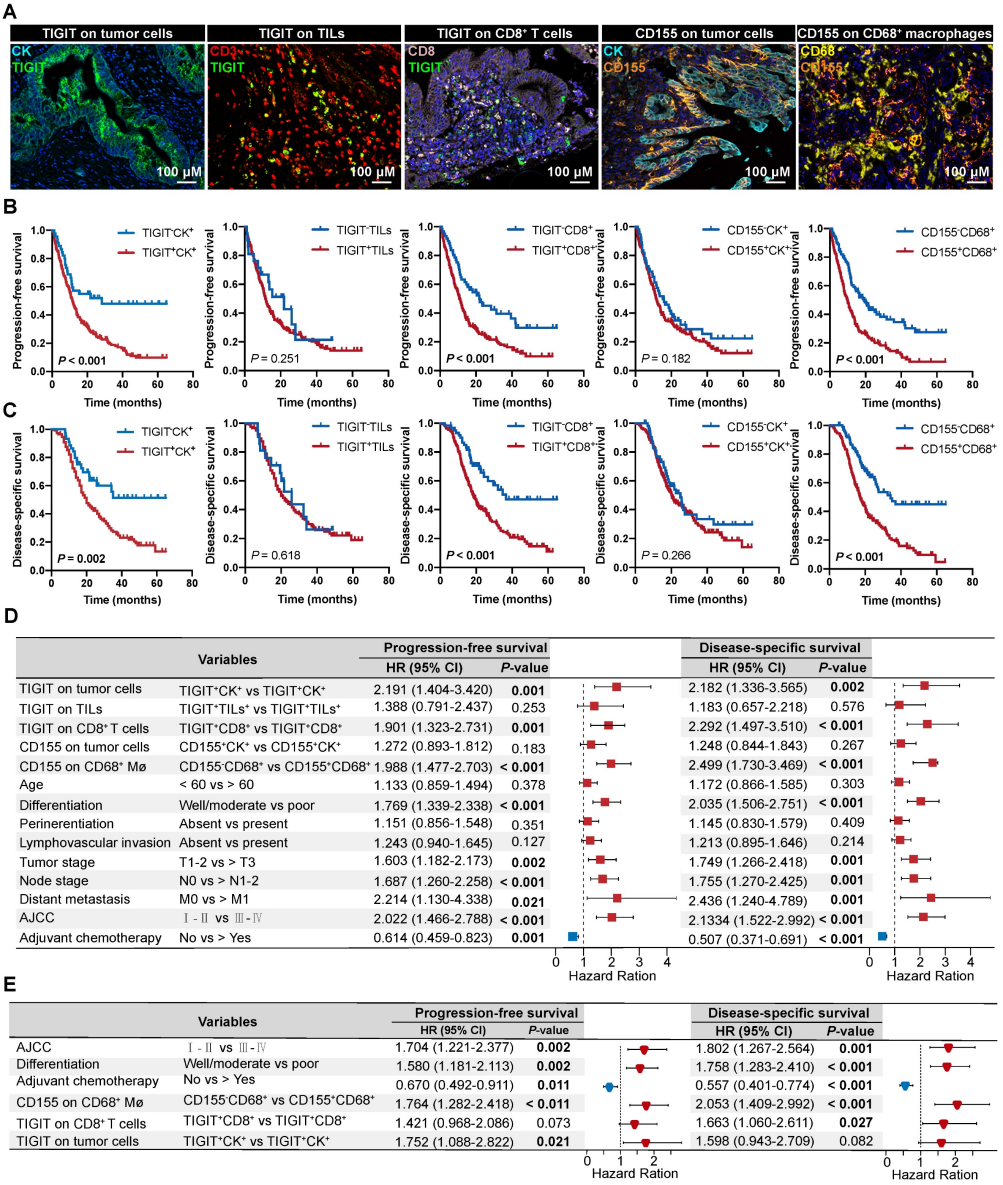
** Prognostic value of TIGIT/CD155 expression in patients with PDAC. (A)** Representative localization composite images of TIGIT expression on tumor cells, TIGIT on TILs, TIGIT on CD8^+^ T cells, CD155 on TCs, and CD155 on macrophages. Scale bar: 100 μm.** (B-C)** Kaplan-Meier survival analysis of PFS and DSS between patients with positive and negative TIGIT/CD155 expression in tumor cells (for both), tumor-infiltrating lymphocytes (TILs) (for TIGIT^+^), CD8^+^ T cells (for TIGIT^+^), and CD68^+^ macrophages (for CD155^+^), respectively. The log-rank [Mantel-Cox] test or the Tarone-Ware test was used for survival analysis. *P* < 0.05 indicates statistically significant. **(D-E)** Univariate and multivariate Cox regression analyses of factors potentially predictive of progression-free survival and disease-specific survival. AJCC, American Joint Committee on Cancer; HR, hazard ratio; CI, confidence interval. *P* values < 0.05 are bolded.

According to the classification of TIGIT and CD155 at different microanatomical subregions based on staining intensity, 272 patients with PDAC who underwent upfront surgery followed by optional adjuvant systemic therapy were stratified into high and low groups and subjected to survival analysis. Representative composite images of the co-expression of TIGIT and CD155 and corresponding markers are shown in **Figure 3A**. Kaplan-Meier curves showed that high TIGIT expression on TCs (TIGIT^+^CK^+^) was significantly correlated with short PFS (*P* < 0.001) and DSS (*P* = 0.002) in the entire cohort. Moreover, patients with positive TIGIT in CD8^+^ T cells (TIGIT^+^CD8^+^) had significantly poor PFS (*P* < 0.001) and DSS (*P* < 0.001) than those with TIGIT^-^CD8^+^ cells. However, TIGIT expression in TILs had no obvious effect on PFS or DSS. In addition, the positive expression of CD155 in TCs had no significant effect on PFS and DSS. However, negative CD155 in CD68^+^ macrophages (CD155^-^CD68^+^) was correlated with favorable PFS (*P* < 0.001) and DSS (*P* < 0.001) (**Figure 3B-C**). Univariate survival analysis showed a similar trend where higher densities of TIGIT on TCs, CD8^+^ T cells, and CD155 on CD68^+^ macrophages were related to inferior PFS and DSS. Tumor well/moderate differentiation, early tumor stage (T1-2), negative nodal metastatic (N0), no distant metastasis (M0), low AJCC stage (I-II), and ACT were associated with high PFS and DSS (**Figure 3D**). Multivariate analysis revealed that TIGIT on TCs or CD155 on macrophages could serve as an independent prognostic biomarker in terms of PFS [hazard ratio (HR) = 1.752, 95% confidence interval (CI), 1.088-2.822; *P* = 0.021 and HR 1.764; 95% CI 1.282-2.418; *P* < 0.001, respectively]. TIGIT on CD8^+^ T cells and CD155 on macrophages were prognostic predictors of improved DSS (HR = 1.663; 95% CI, 1.060-2.611; *P* = 0.027 and HR 2.053; 95% CI, 1.409-2.992; *P* < 0.001, respectively], independent of AJCC stages, differentiation grades, and ACT (**Figure 3E**).

### Correlation between ICs phenotypes and distinct TIGIT/CD155-positive subtypes

**Figure 4:**
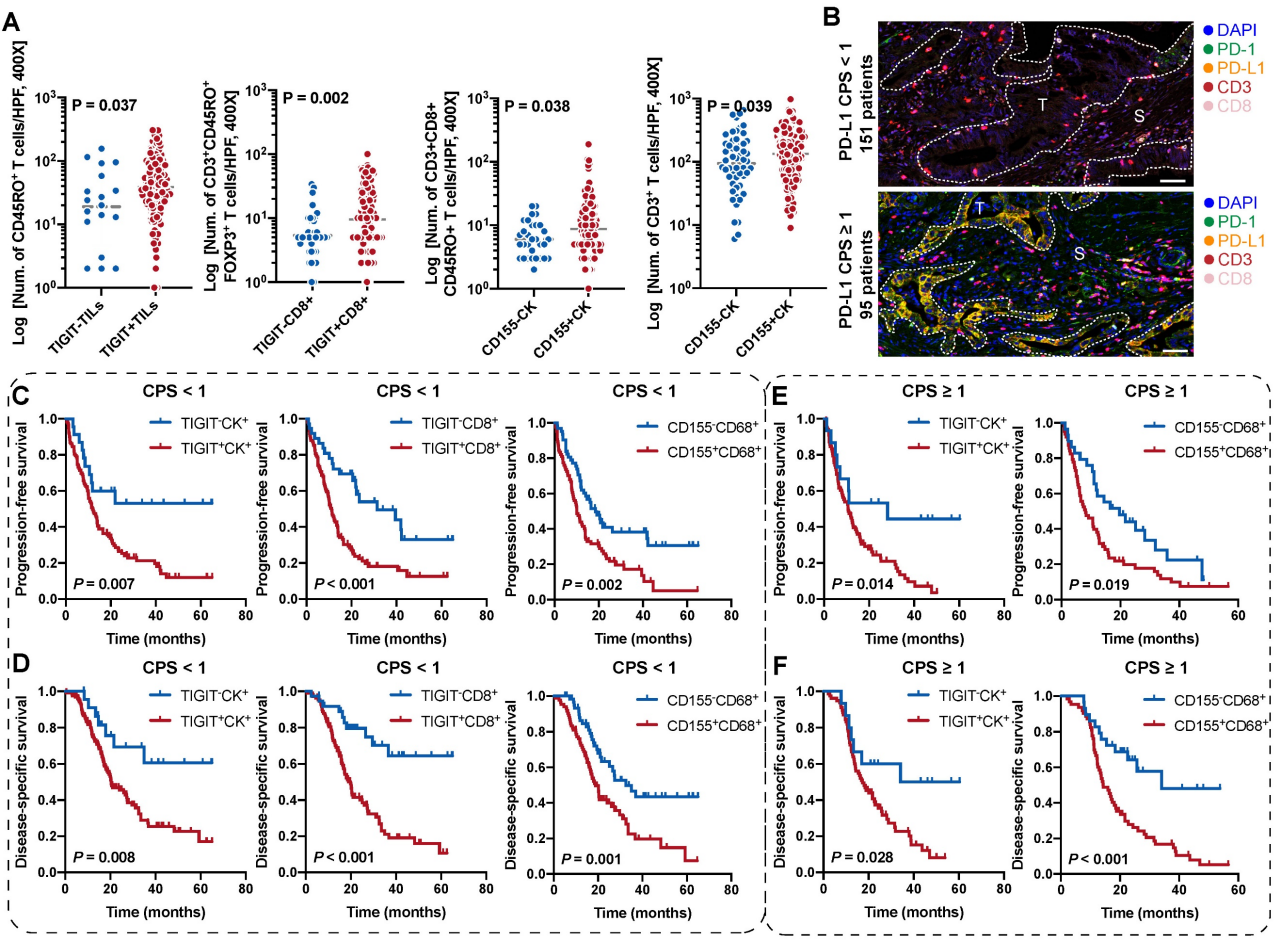
** Survival analysis of the TIGIT/CD155-positive phenotypes in patients with PDAC stratified by PD-L1 status. (A)** Comparison analysis of the densities of distinct immune cells grouped by the expression of TIGIT on TILs and CD8^+^ T cells and the expression of CD155 on tumor cells. Quantitative correlation determined by Mann-Whitney *U* test. *P* values < 0.05 are bolded.** (B)** Presentative mIHC images of PD-L1 CPS < 1 and CPS ≥ 1 according to PD-L1 expression. **(C-F)** Kaplan-Meier curves showing the progression-free survival (PFS) and disease-specific survival (DSS) according to the expression of TIGIT/CD155 in the patients with PDAC group by PD-L1 status. PFS **(C)** and DSS **(D)** under PD-L1 CPS < 1. PFS **(E)** and DSS **(F)** under PD-L1 CPS ≥ 1. Log-rank [Mantel-Cox] test or Tarone-Ware test was used for survival analysis. *P* < 0.05 indicates statistically significant.

To evaluate the enrichment and distribution of ICs in the five distinct TIGIT/CD155-positive subtypes, we observed that CD3^+^ T cells with the highest spatial density among all ICs, followed by CD68^+^ macrophages, CD8^+^ T cells were relatively few, the numbers of CD3^+^CD8^+^CD45RO^+^, CD3^+^CD8^+^GB^+^, and CD3^+^CD45RO^+^FOXP3^+^ T cells were small in PDAC tissues (**Figure S4**). When grouped by TIGIT^+^CK^+^, no differences were observed among these phenotypes (**Figure S5A**). However, TIGIT^+^ TILs were correlated with high densities of CD45RO^+^ T cells; TIGIT^+^CD8^+^ T cells were associated with high infiltration of CD3^+^CD45RO^+^FOXP3^+^; CD155^+^CK^+^ were significantly related to high densities of CD3^+^ and CD3^+^CD8^+^CD45RO^+^ T cells (**Figure 4A, Figure S5B-D**). But few differences were observed in the ICs density of these immune cells between CD155-negative and -positive macrophages (**Figure S5E**). Phenotypes with statistical differences in densities were subjected to Pearson correlation analysis, which showed low correlations, albeit with the suggestion that TIGIT- or CD155- positive cells were associated with T-cell exhaustion (**Figure S6A**). Briefly, these results indicate that the TIGIT/CD155-mediated immunosuppression axis was related to low immunogenic phenotypes and suppressive microenvironment.

### Prognostic values of the TIGIT- and CD155-positive phenotypes stratified by PD-L1 status

Combinatorial blockade of TIGIT and PD-L1 shows promising efficacy for antitumor immunotherapy 23, 24. We speculated whether the stratification efficacy of TIGIT for patients with PDAC can be further modified by PD-L1. According to the CPS for PD-L1 expression, CPS < 1 was 61.38% (151/246) while 38.62% (95/246) of patients presented a CPS ≥ 1(**Figure 4B**). The densities of TIGIT/CD155-positive ICs in (PD-L1, CPS ≥ 1) were no different from those in (PD-L1, CPS < 1) (**Figure S6B**). In the (PD-L1, CPS < 1) subgroup, patients with TIGIT^+^CK^+^, TIGIT^+^CD8^+^, or CD155^+^CD68^+^ had poorer PFS and DSS than those of their negative counterparts (**Figure 4C-D**). Interestingly, patients with higher densities of TIGIT^+^CK^+^ or CD155^+^CD68^+^ had lower PFS and DSS in the (PD-L1, CPS ≥ 1) subgroup than those of their negative counterparts (**Figure 4E-F**). However, no differences were observed in PFS and DSS among patients with TIGIT^+^TILs^+^, TIGIT^+^CD8^+^, and CD155^+^CK^+^ regardless of PD-L1 status (**Figure S6C**). In addition, patients with TIGIT/CD155-positive subtypes had an increased risk of death both in CPS < 1 and CPS ≥ 1 of PD-L1 (**Figure S7A-B**). Collectively, our data demonstrate the potential prognostic value of TIGIT/CD155, which may be correlated with PD-L1 status. Therefore, it is necessary to explore their biological roles in future research.

### TIGIT and CD155 co-expression defines inferior outcomes and identifies responders to ACT

**Figure 5:**
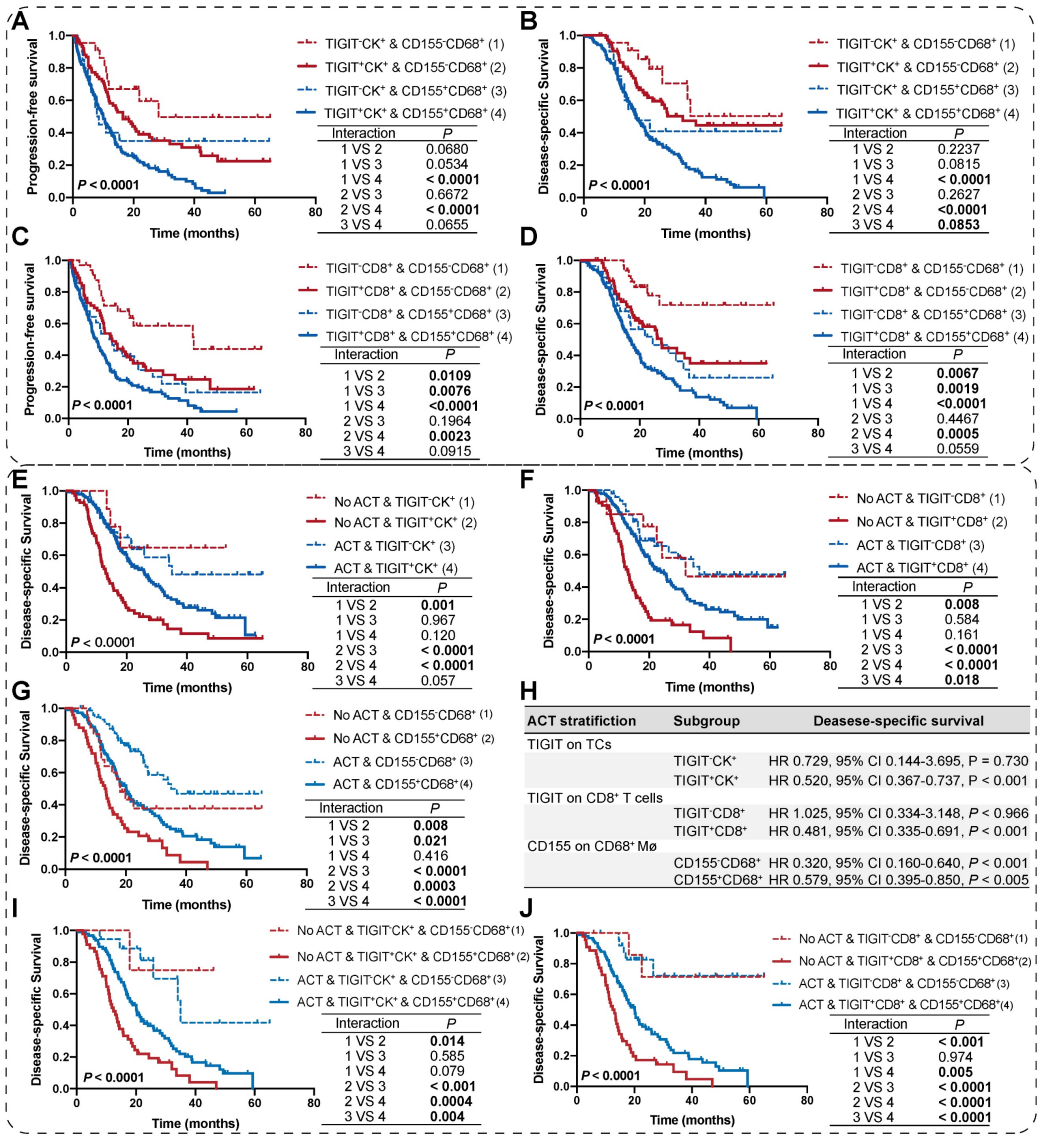
**Survival analysis of the co-expression of TIGIT/CD155 subgroups and their prognostic prediction of response to adjuvant chemotherapy. (A-D)** PFS and DSS are stratified by combining positive/negative TIGIT on TCs or CD8^+^ T cells and positive/negative CD155 on macrophages. **(E-G)** DSS of ACT-treated patients with positive/negative TIGIT on TCs or CD8^+^ T cells and positive/negative CD155 on macrophages. **(H)** Multivariate Cox analysis of independent predictors for distinguishing potential responders to ACT. **(I-J)** DSS of ACT-treated patients with co-existence of TIGIT and CD155 subgroups. Log-rank [Mantel-Cox] test or Tarone-Ware test was used for survival analysis. *P* < 0.05 indicates statistically significant.

As TIGIT on TCs or CD8^+^ T cells and CD155 on macrophages individually signified poor PFS and DSS, we analyzed the presence of potential combined subtypes that have not yet been identified and could serve as biomarkers for pretreatment stratification of patients with PDAC. Patients with TIGIT^+^CK^+^ and CD155^+^CD68^+^ had PFS and DSS significantly poorer than those with TIGIT^-^CK^+^ and CD155^-^CD68^+^ (**Figure 5A-B**). Similar results were observed for patients with TIGIT^+^CD8^+^ and CD155^+^CD68^+^ who had PFS and DSS poorer than those with TIGIT^-^CD8^+^ and CD155^-^CD68^+^ (**Figure 5C-D**).

We then evaluated the potential clinical efficiency of combining any two phenotypes to identify responders who are most likely to benefit from ACT. In this retrospective cohort, 70.22% (191/272) of patients with PDAC received ACT. First, we found that the risk scores for patients with PDAC were positively correlated with TIGIT/CD155-positive subtypes, regardless of ACT treatment (**Figure S7C-D**). Second, survival analysis confirmed that TIGIT^-^CD8^+^ T cells (rather than TIGIT^+^ TCs), and CD155^-^ macrophages significantly improved the DSS of PDAC patients who received ACT. Similarly, patients with TIGIT^-^CK^+^, TIGIT^-^CD8^+^, or CD155^-^CD68^+^ responded more strongly to ACT than their positive counterparts (**Figure 5E-G**). However, multivariate Cox regression analysis revealed that only TIGIT on TCs and CD8^+^ T cell subgroups could be predictors of superior response to ACT in PDAC (**Figure 5H**). Notably, under the precondition of PDAC patients received ACT, those with spatial coexistence of TIGIT^+^CK^+^ or TIGIT^+^CD8^+^ and CD155^+^CD68^+^ exhibited poorer DSS compared to the cases with spatial non-coexistence of the counterparts (TIGIT^-^CK^+^ or TIGIT^-^CD8^+^ and CD155^-^CD68^+^) (3 vs 4) (**Figure 5I-J**), suggesting that the spatial co-expressed of TIGIT and CD155 may contribute to enhanced resistance to ACT in a patient with PDAC. Overall, our findings demonstrate that the co-occurrence of TIGIT and CD155 in PDAC tumor microenvironment weakens the favorable outcomes for PDAC patients regardless of whether treated with ACT or not (1 vs 2; 3 vs 4), highlighting the need for subgroup analysis strategies to stratify patients for more rational and effective treatments.

## Discussion

Targeting emerging immune checkpoint pathways has revolutionized cancer immunotherapy 25. However, the modest incremental clinical benefits of immune checkpoint blockade are only applicable to a fraction of patients with PDAC. Further comprehensive and in-depth investigations of spatial immune features and prognostic values of unidentified IC phenotypes in the PDAC ecosystem are warranted. In this study, we investigated the TIGIT/CD155 axis, a high-profile immune-suppression checkpoint, analyzed its expression patterns and clinical significance, and investigated the relationship between TIGIT/CD155-positive subgroups and multiple distinct immunologic phenotypes using mIHC in three diverse panels constructed from 272 archived PDAC specimens.

mIHC technologies play an irreplaceable role in simultaneously profiling the spatial distribution metric, analyzing the co-expression of multifunctional ICs *in situ*, and unearthing potential valuable immune phenotypes as compared with conventional IHC 26-29. Taking advantage of this robust platform, we captured TIGIT-positive cells that closely colocalized with CD3^+^ T-cell subgroups (tightly restricted to lymphocytes) in the stromal compartments, confirming its crucial role as an inhibitory immunoreceptor for compromising acute T-cell function 11, 24. However, mIHC has some limitations. Excessive sensitivity caused by tyramide signal amplification-based signal amplification of tyramide fluorophores (particularly in the Opal 480 channel) directly leads to incorrect identification of cell phenotypes (low specificity) using a phenotyping setting based on the Infrom system. The staining order of multiple markers is another challenge, which determines the sensitivity to antigens and spectral bleed-through 29. Therefore, we performed optimization and validation more than once to avoid these issues before the formal experiments, although all immune variables were manually evaluated in this study.

The TME is a complex ecosystem featuring an abundance of low-immunogenic components including exhausted ICs, multi-faceted desmoplastic stroma, and cancer cells that create a hostile environment for the activation of T cell-mediated immune response and hamper the efficacy of immnuotherapy 30, 31. Various strategies are being developed to reverse endogenous immunodeficiencies or reinvigorate tumor-infiltrating effector CD8^+^ T cells to prime PDAC-specific immunity. For example, targeting co-inhibitory pathways using anti-PD-L1 antibody pembrolizumab 32, and anti-CTLA-4 antibody ipilimumab 33 and tremelimumab 34, or targeting co-stimulatory targets OX40 35, 41BB 36, and CD40 37 have considerable potential against fully-effective and rejecting PDAC albeit with underwhelming clinical efficiency at present. We revealed that the PDAC TME featured high heterogeneity and poor immunogenicity, consistent with those of previous studies. Specifically, we identified various ICs phenotypes, including CD3^+^ T cells and CD68^+^ macrophages with high densities, CD8^+^ T cells, CD45RO^+^ T cells, and FOXP3^+^ regulatory T cells with relatively low densities, and a few infiltrations of CD3^+^CD8^+^CD45RO^+^, CD3^+^CD8^+^GB^+^, and CD3^+^CD45RO^+^FOXP3^+^ T cells. Notably, TIGIT and CD155 were mainly located on CD8^+^ T cells and CD68^+^ macrophages, respectively. The density and quantity of TIGIT^+^CD8^+^ T cells within the stromal compartments of PDAC are not high, which indirectly shows that the PDAC TME is immune-dormant. Paradoxically, a recent study demonstrated that TIGIT was expressed on 53% and 28% of CD8^+^ and CD4^+^ T cells, respectively, within PDAC tumor-infiltrating lymphocyte populations 38. TIGIT ligand CD155 was abundantly expressed on immunosuppressive M2-like macrophages 39, which is consistent with our findings. Additionally, high levels of coexpression of TIGIT and PD-1 are observed on CD8^+^ and CD4^+^ T cells within the PDAC microenvironment 38. Those results suggest that TIGIT/CD155 axis may have the potential to modulate the immunosuppressive microenvironment, and further investigation of its biological role and clinical implications is warranted for precision immunotherapy of patients with PDAC.

Aberrant expression of distinct cell-surface antigens involved in coinhibitory pathways induced by TIGIT/CD155, PD-1/PD-L1, and CTLA-4, aggravates the accumulation of exhausted CD8^+^ T cells and hampers the efficacy of traditional immunotherapy 40. Therefore, identifying potential combinatorial strategies for the priming and activation of T-cell functionality is the major goal of most immunotherapies. The TIGIT/CD155 axis is a critical driver of immune evasion in various malignancies, which has drawn widespread attention in antitumor immunity; however, this has not been extensively studied in the context of PDAC, particularly for clinical prognostic analysis based on large cohorts of patients with PDAC. Therefore, this study systematically analyzed the clinicopathological relationship and clinical significance of TIGIT and CD155 in 272 archived PDAC specimens. We did not observe significant correlations between clinicopathological features and distinct TIGIT/CD155-positive phenotypes, except for TIGIT positivity on TCs and CD8^+^ T cells that were significantly related to high AJCC and N1-2 stages, respectively. CD155 on macrophages was positively associated with head and neck localization. Notably, we found that patients with high densities of TIGIT expression in TCs, rather than CD155 expression in TCs, had poor prognoses in patients with PDAC. Previous studies have demonstrated that TIGIT expression is elevated in various cancers, including pancreatic cancer 13, 41, bladder cancer 42, and cervical cancer 43. Acquired TIGIT expression in TCs with mantle cell lymphoma relapse enhances the interaction of TIGIT^+^ TCs with monocyte CD155/PVR 44, suggesting that a rational strategy could be considered for targeting TIGIT to enhance the anti-tumor immunotherapy efficacy. Unexpectedly, we discovered that TIGIT was expressed more frequently in TCs with membranes and cytoplasmic staining than in adjacent normal tissues. Moreover, TIGIT expression in CD8^+^ T cells, or CD155 expression in macrophages, was associated with inferior outcomes in patients with PDAC, which is consistent with the aforementioned evidence. Remarkably, the coexistence of TIGIT^+^ TCs or CD8^+^ T cells with CD155^+^ macrophages exacerbates the mortality of patients with PDAC. PD-L1 expression has been used as a robust predictor to guide the application of anti-PD-1 therapy 45, 46. Our results revealed that patients with TIGIT^+^CK^+^ or CD155^+^CD68^+^ were associated with poor prognoses that may relate to PD-L1 status. However, the prognostic value of TIGIT^+^CD8^+^ cells was significant only in the (PD-L1, CPS < 1) subgroup. The synergistic crosstalk of various ICs phenotypes orchestrated by the TIGIT/CD155 axis is indispensable for the amplifying of the immunosuppressive effect. Unfortunately, only a few immunophenotypes of interest have been observed, which are significantly related to TIGIT^+^ or CD155^+^ cells in the PDAC TME, such as CD3^+^CD8^+^GB^+^ T cells positively correlated with TIGIT^+^ TILs but negatively associated with CD155^+^ TCs, suggesting a spatial perturbation of CTLs modulated by the TIGIT/CD155 axis.

Combinatorial treatment through immunotherapy synergized with ACT may promote tumoricidal activity by enhancing T-cell priming and expanding effector T-cell proliferation 47, 48. In gastric cancer, peritumoral TIGIT^+^CD20^+^ B cell infiltration is associated with poor prognosis and also benefits more from ACT 49. High TIGIT expression has a favorable response to ACT and anti-PD-L1 immunotherapy 42. Intriguingly, in the context of PDAC patients who received ACT, patients with immunosuppressive features (TIGIT^+^CK^+^, TIGIT^+^CD8^+^, or CD155^+^CD68^+^) were more likely to be resistant to ACT. Furthermore, any combination of these features exacerbates the outcomes of patients with PDAC, who receive ACT. These results highlight the prospects of targeting the TIGIT/CD155 axis in combination with ACT for rejecting PDAC and underscore that accurate prognostic evaluation is critical for selecting appropriate treatments for patients with PDAC.

This study has some strengths; however, inherent limitations still exist. First, all PDAC specimens were retrospectively obtained from an independent cohort, suggesting that further validation in large prospective external cohorts is required. Additionally, intra-tumoral heterogeneity is inevitable when using TMA. Finally, spatial proximity was not analyzed because of the misrecognition of ICs and infiltrated red blood cells in TILs of PDAC using a phenotyping algorithm.

## Conclusion

Our results highlight the potential prognostic value of the TIGIT/CD155 axis in patients with PDAC, which merits future consideration to combine these immune checkpoints for personalized immunotherapy against PDAC.

## Supplementary Material

Supplementary figures and tables.

## Abbreviations

PDAC: pancreatic ductal adenocarcinoma
TIGIT: T cell immunoreceptor with immunoglobulin and ITIM domain
TCs: tumor cells
ICs: immune cells
ACT: adjuvant chemotherapy
TILs: tumor-infiltrating lymphocytes
TME: tumor microenvironment
ICIs: immune checkpoint inhibitors
PD-L1: programmed cell death protein 1
CTLA-4: cytotoxic T-lymphocyte antigen 4
mIHC: multiplex immunohistochemistry
FOXP3: forkhead box P3
GB: granzyme B
PFS: progression-free survival
DSS: disease-specific survival
ROIs: regions of interest
TMA: tissue microarray
CPS: combined positive score
TC-ISA: TIGIT/CD155-immunosuppression axis
PP-ISA: PD-1/PD-L1 immunosuppression axis
TCGA: The Cancer Genome Atlas
AJCC: American Joint Committee on Cancer
HR: hazard
CI: 95% confidence interval
HPF: high-power field
