# Supplementary material for: The spatial coexistence of TIGIT/CD155 defines poorer survival and resistance to adjuvant chemotherapy in pancreatic ductal adenocarcinoma
Source: Theranostics. 2023 Aug 18;13(13):4601–14. doi: 10.7150/thno.86547 (PMC10465224; doi:10.7150/thno.86547)
Supplement: Supplementary file 1 — Supplementary figures and tables. [file thnov13p4601s1.pdf]

Supplementary Material

The spatial coexistence of TIGIT/CD155 defines poorer survival and resistance to adjuvant chemotherapy in pancreatic ductal adenocarcinoma

Heng Ma[1]*, Xianlong Chen[1]*, Shengwei Mo[1], Xinxin Mao[1], Jingci Chen[1], Yilin Liu[1], Zhaohui Lu[1], Shuangni Yu[1†], Jie Chen[1†]

Affiliations:

[1]Department of Pathology, Peking Union Medical College Hospital, Peking Union Medical College and Chinese Academy of Medical Science, Beijing, 100730, China

*These authors contributed equally to this work.

[†]These authors jointly supervised this work.

[†]**Corresponding Author:**

Jie Chen, Department of Pathology, Peking Union Medical College Hospital, Peking Union Medical College and Chinese Academy of Medical Science, Beijing, 100730, China. E-mail: chenjie@pumch.cn. Orcid ID: 0000-0002-2658-9525.

Shuangni Yu, Department of Pathology, Peking Union Medical College Hospital, Peking Union Medical College and Chinese Academy of Medical Science, Beijing, 100730, China. E-mail: yushn@pumch.cn. Orcid ID: 0000-0002-3745-1097.

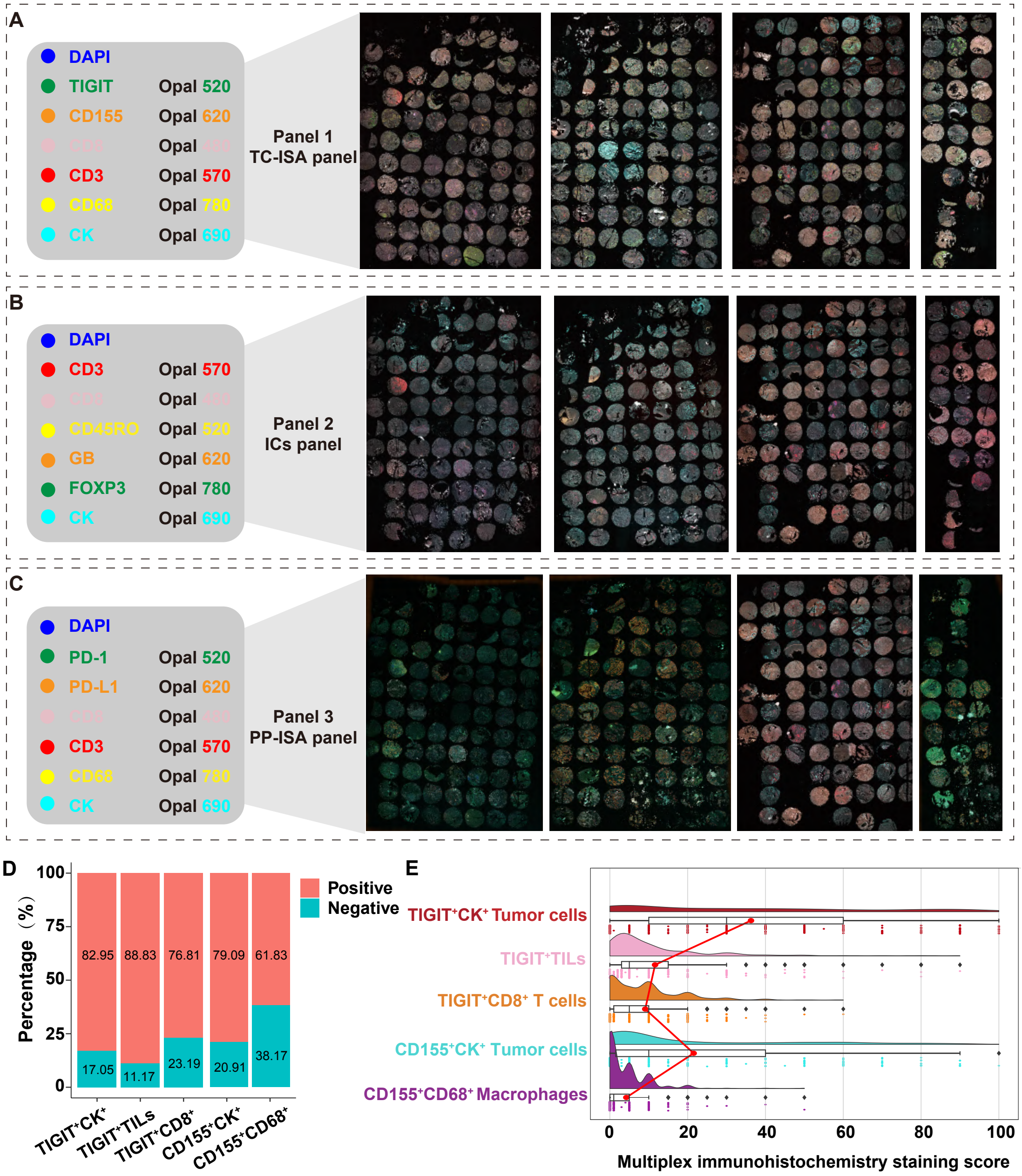

Supplementary Figure 1. Overview of color schemes and the raw TMA images. (A) TC-ISA, TIGIT/CD155 immunosuppression checkpoints. **(B)** ICs, immune cells. **(C)** PP-ISA, PD-1/PD-L1 immunosuppression checkpoints. **(D)** Positive expression rates for five identified phenotypes associated with TIGIT/CD155. **(E)** Raincloud plots display the distribution of TIGIT/CD155-positive phenotypes.

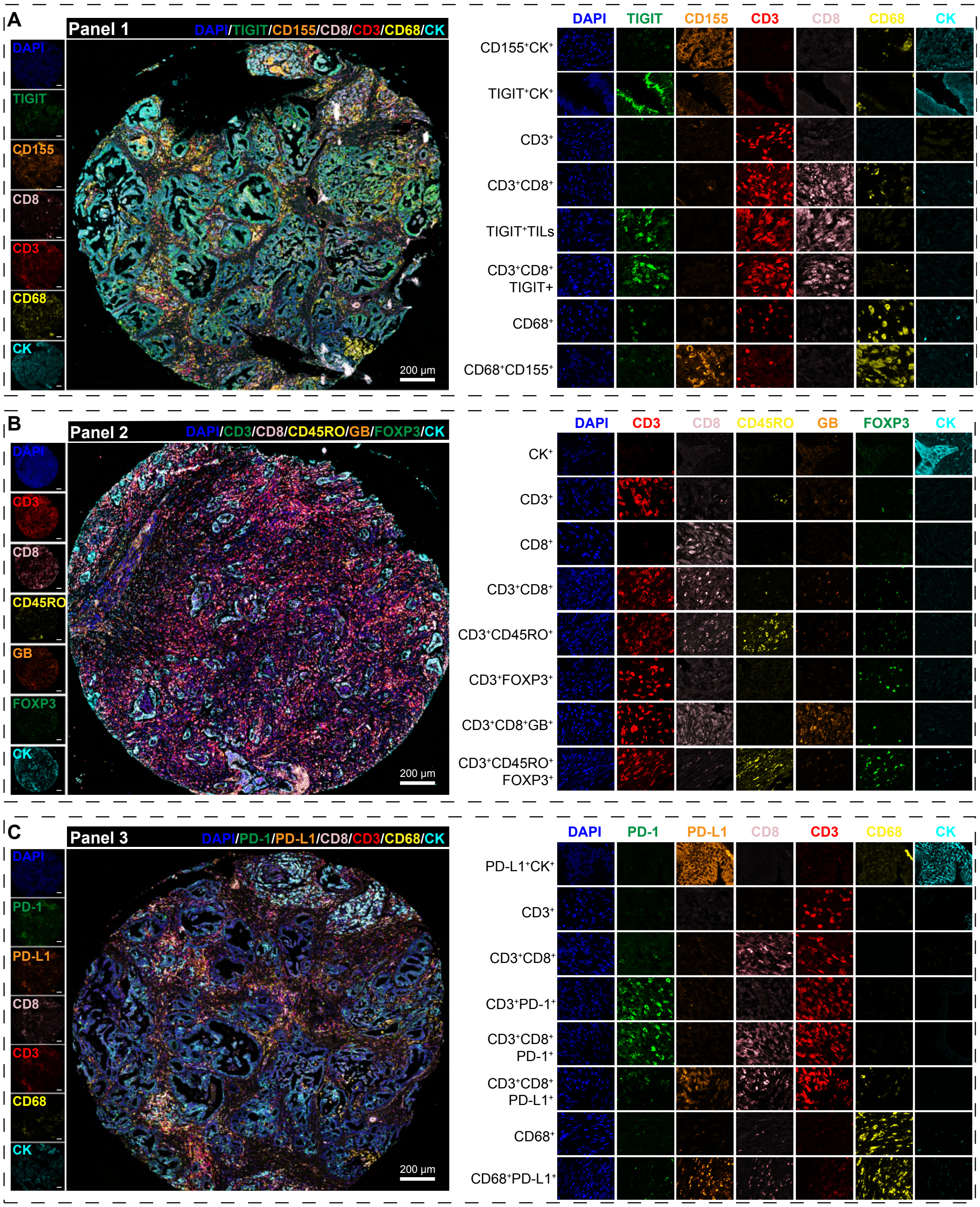

Supplementary Figure 2. Representative single or colocalization immune markers-stained images from 291 PDAC TMA core. (A) TIGIT-expressing malignant cells ($TIGIT^{+}CK^{+}$), TILs expressing TIGIT ($TIGIT^{+}$ TILs), cytotoxicity T lymphocytes (CTLs) expressing TIGIT ($TIGIT^{+}CD3^{+}CD8^{+}$), TCs expressing CD155 ($CD155^{+}CK^{+}$), and macrophages expressing CD155

($CD155^+CD68^+$) in the TIGIT/CD155 immunosuppression axis panel. Scale bar: 50 μm. **(B)** The immune variables such as CTLs ($CD3^+CD8^+$), memory T lymphocytes ($CD3^+CD45RO^+$), regulatory T lymphocytes ($CD3^+FOXP3^+$), activated CTLs ($CD3^+CD8^+GB^+$), and memory/regulatory T lymphocytes ($CD3^+CD45RO^+FOXP3^+$) in immune cells panels. Scale bar: 50 μm. **(C)** PD-L1-expressing TCs ($PD\text{-}L1^+CK^+$), PD-L1-expressing macrophages ($PD\text{-}L1^+CD68^+$), antigen-experienced CTLs ($PD\text{-}1^+CD3^+CD8^+$), and CTLs expressing PD-L1 ($PD\text{-}L1^+CD3^+CD8^+$) in PD-1/PD-L1 immunosuppression axis panels. Scale bar: 50 μm.

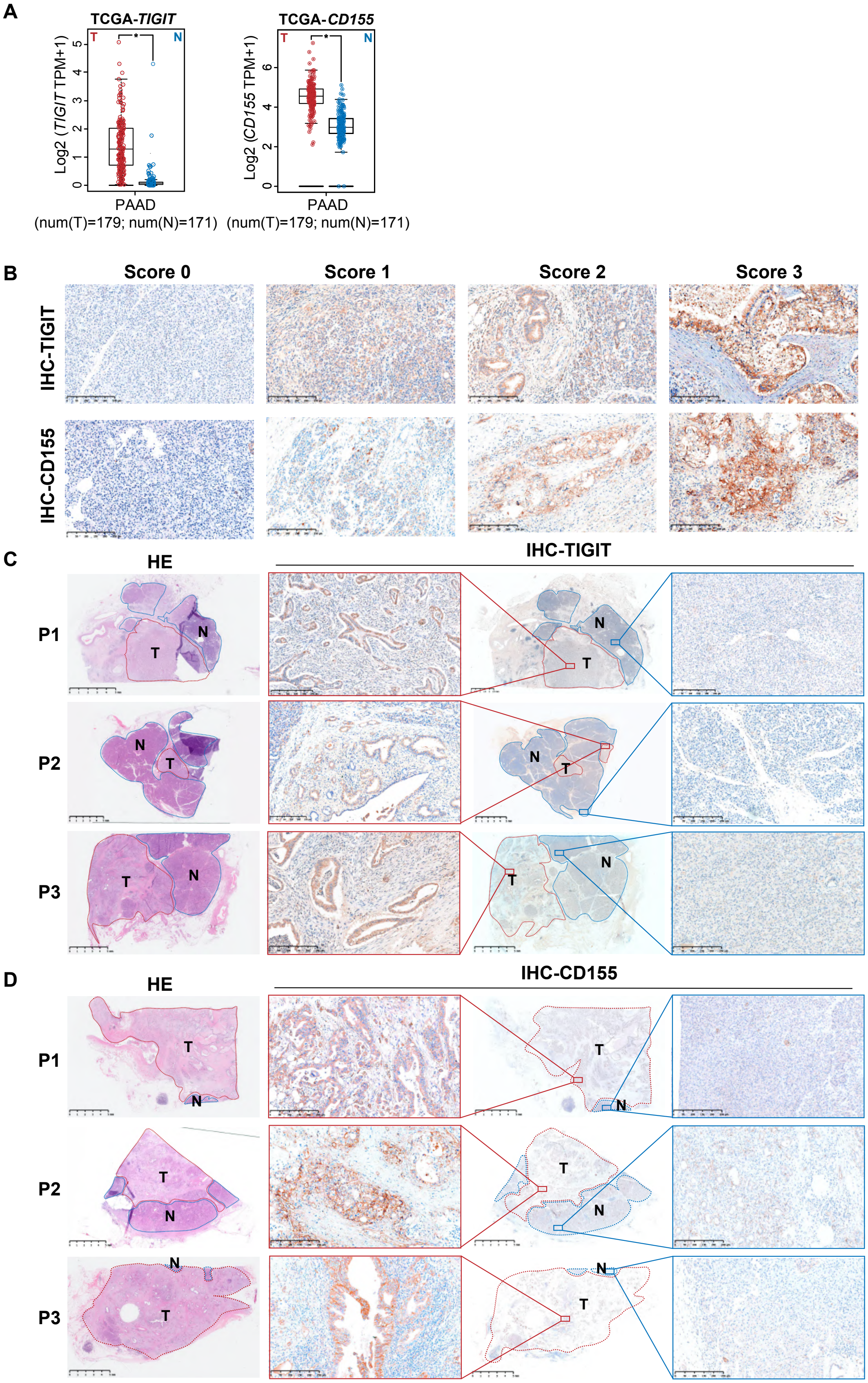
A
TCGA-TIGIT
TCGA-CD155
T
N
*
Log2 (TIGIT TPM+1)
Log2 (CD155 TPM+1)
PAAD
(num(T)=179; num(N)=171)
PAAD
(num(T)=179; num(N)=171)
B
Score 0
Score 1
Score 2
Score 3
IHC-TIGIT
IHC-CD155
C
HE
IHC-TIGIT
P1
P2
P3
D
HE
IHC-CD155
P1
P2
P3

Supplementary Figure 3. The expression of TIGIT and CD155 in PDAC was determined by TCGA datasets and immunohistochemistry. (A) The mRNA levels of *TIGIT* and *CD155* in normal control and PDAC patients were analyzed from the TCGA datasets (http://cancergenome.nih.gov/). T: tumor (n = 179); N: pancreatic normal tissues (n = 171). **(B)** The representative intensity images for each IHC score of TIGIT and CD155 staining in PDAC tissues were shown. Score 0, 1, 2, 3 denotes negative, weak, medium, and firm determined by H-score. **(C-D)** Immunohistochemical analysis was performed on primary PDAC tissues and matched adjacent noncancer tissues to investigate the expression of TIGIT and CD155 on whole tissue sections. HE, Hematoxylin and Eosin; P, Patient; N, normal pancreatic cells; T, Tumor cells.

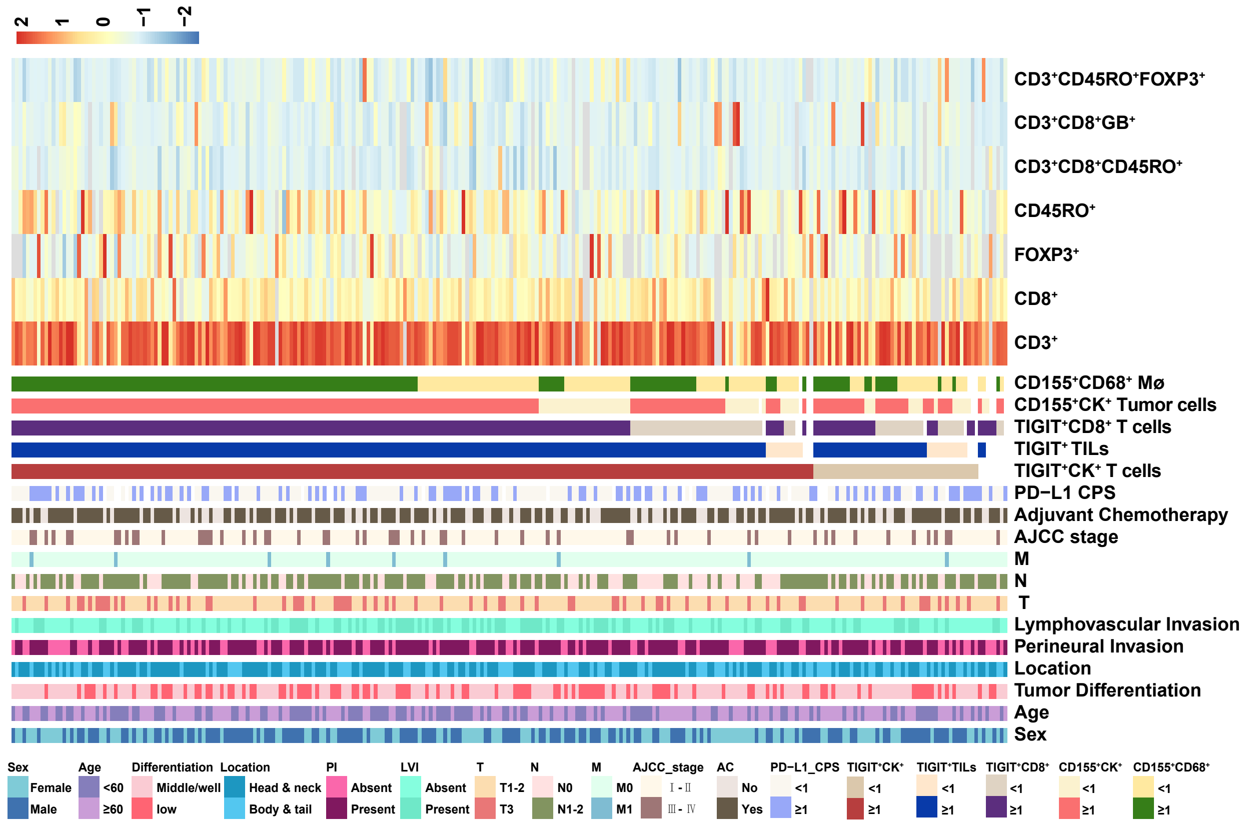

Supplementary Figure 4. Heatmap displays the proportions and overlap between immune cells and clinicopathologic features associated with TIGIT and CD155 phenotypes. Mø, macrophages; TILs, tumor infiltration lymphocytes; CPS, combined positive scores; T, tumor stage; N, node stage; M, distant metastasis; AC, adjuvant chemotherapy; PI, perineural invasion; LVI, and lymphovascular invasion.

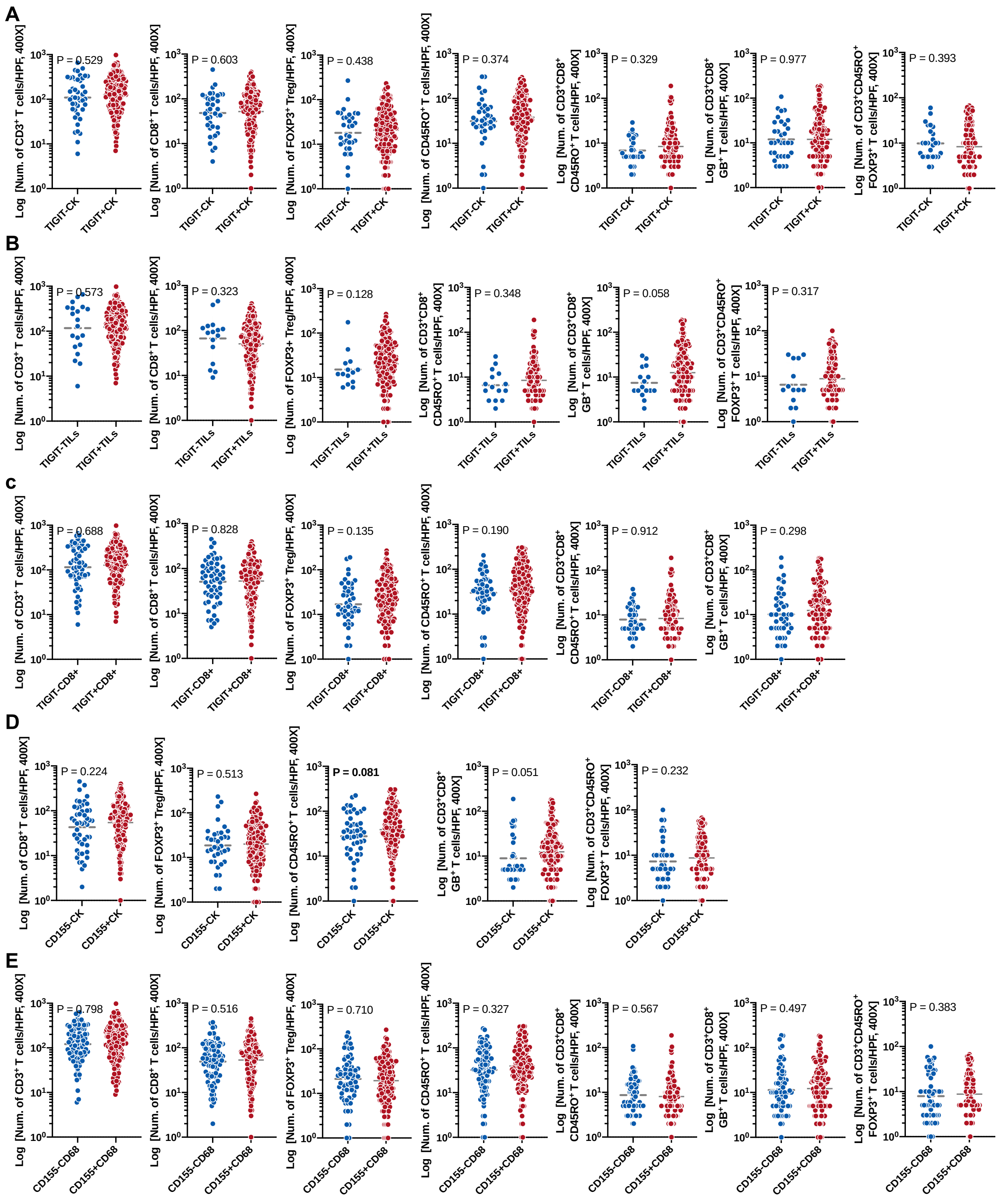

Supplementary Figure 5. Comparison analysis of the densities of immune cells across distinct TIGIT/CD155-positive subgroups. (A-C) Grouped by the expression of TIGIT on tumor cells **(A)**, TILs **(B)**, and CD8^{+} T cells **(C)**. **(D-E)** Grouped by the expression of CD155 on tumor cells

(D), and CD68$^+$ macrophages **(E)**. Quantitative correlation determined by Mann-Whitney *U* test. *P* values <0.05 are bolded. HPF, high-power field.

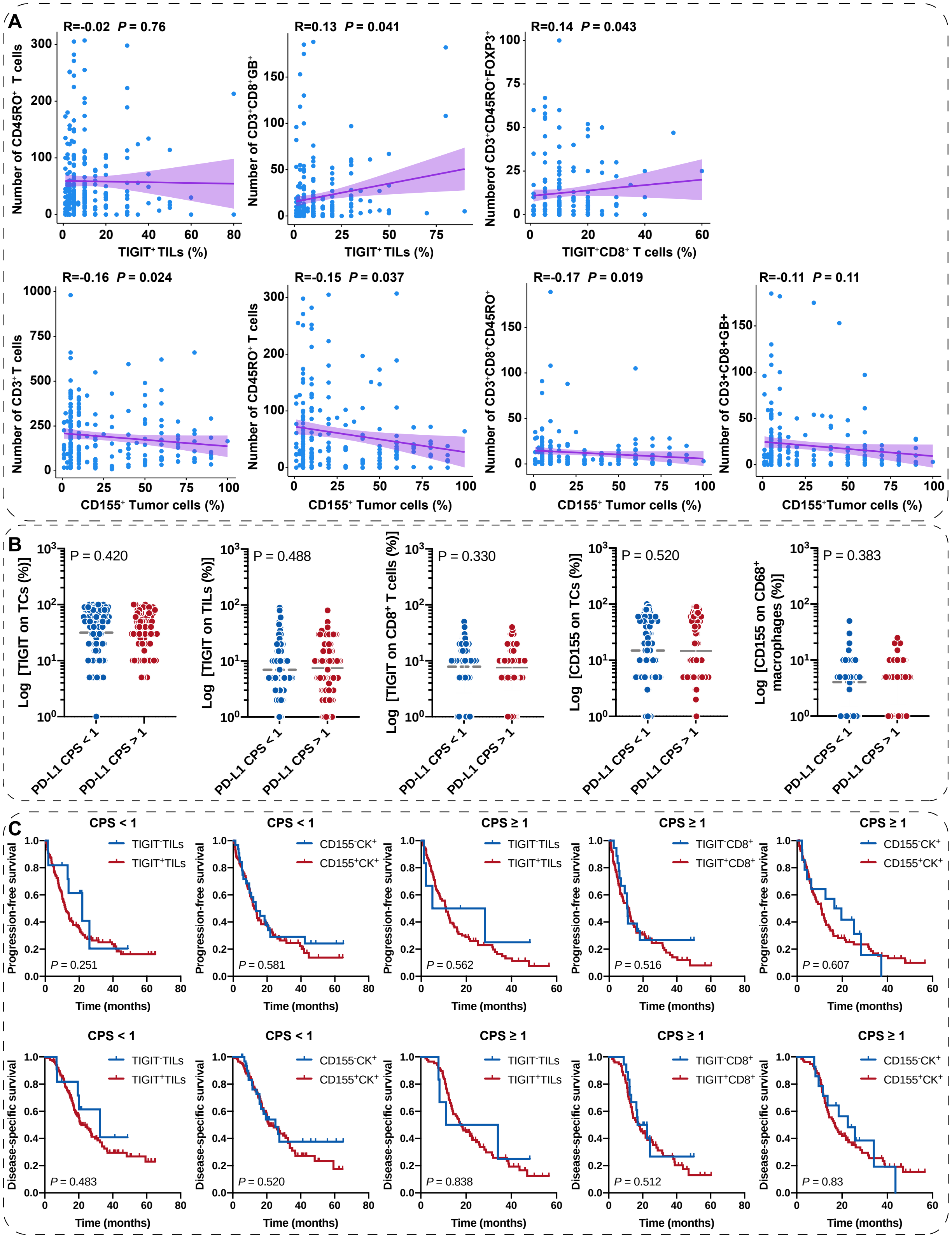

Supplementary Figure 6. Correlation analysis across immune cells and survival analysis of the TIGIT/CD155-positive phenotypes in patients with PDAC stratified by PD-L1 status. (A) Spearman's correlation analysis between the mentioned immune cells and TIGIT on TILs (or $CD8^+$ T cells) or CD155 on TCs. **(B)** The scatter plot presents the densities of TIGIT on TCs, TILs, $CD8^+$ T cells, and CD155 on TCs or macrophages grouped by PD-L1 status. **(C)** Kaplan-Meier curves showing the progression-free survival (PFS) and disease-specific survival (DSS) according to the TIGIT/CD155-positive phenotypes in the patients with PDAC group by PD-L1 status. PFS (up panel) and DSS (bottom panel) under PD-L1 CPS < 1 and CPS ≥ 1. CPS, combined positive score; TCs, tumor cells; TILs, tumor-infiltrating lymphocytes. Quantitative correlation determined by Mann-Whitney *U* test.

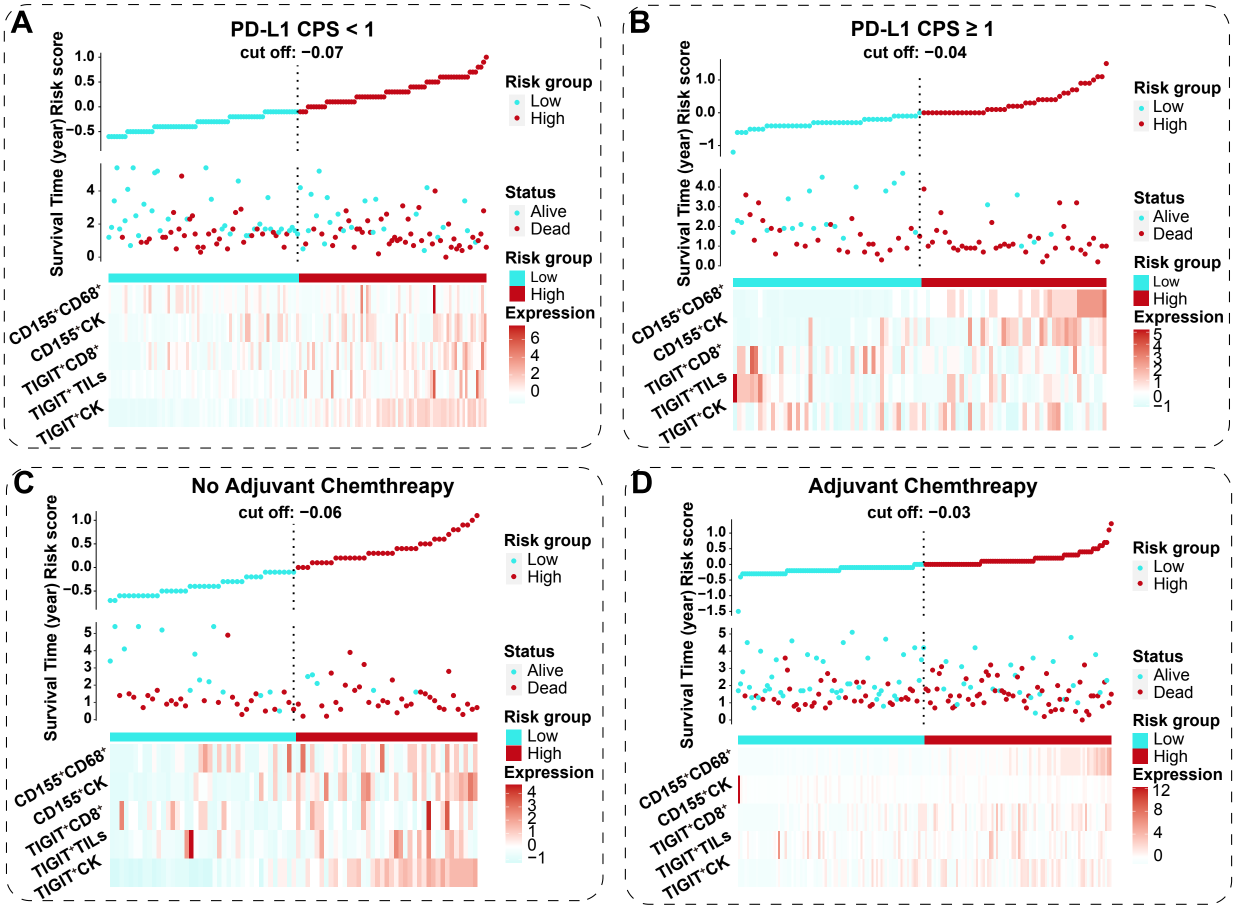

Supplementary Figure 7. Triptych of risk scores displayed the distribution of risk score, survival status, and the abundance of TIGIT/CD155-positive phenotypes. (A-B) Grouped by PD-L1 CPS < 1 or CPS ≥ 1. **(C-D)** Grouped by adjuvant chemotherapy.

Supplementary Table 1. Details of antibodies used for multiple immunohistochemistry.

Group	Antibodies	Source	Catalog number	Dilution	Fluorophore	Color
	Rabbit monoclonal TIGIT	Abcam	ab243903	1/200	Opal 520	green
	Rabbit monoclonal CD155	Cell Signaling Technology	81254S	1/200	Opal 620	brown
Panel 1	Rabbit monoclonal CD3	Abcam	ab16669	1/150	Opal 570	red
	Rabbit monoclonal CD8	Abcam	ab217344	1/200	Opal 480	pink
	Rabbit monoclonal CD68	Cell Signaling Technology	76437S	1/600	Opal 780	yellow
	Mouse monoclonal CK	Cell Signaling Technology	4545T	1/500	Opal 690	cycan
Panel 2	Rabbit monoclonal PD-1	Abcam	ab137132	1/500	Opal 520	green
	Rabbit monoclonal PD-L1	Cell Signaling Technology	13684S	1/200	Opal 620	brown
	Rabbit monoclonal CD3	Abcam	ab16669	1/150	Opal 570	red
	Rabbit monoclonal CD8	Abcam	ab217344	1/200	Opal 480	pink
	Rabbit monoclonal CD68	Cell Signaling Technology	76437S	1/600	Opal 780	yellow
	Mouse monoclonal CK	Cell Signaling Technology	4545T	1/500	Opal 690	cycan
	Rabbit monoclonal CD3	Abcam	ab16669	1/150	Opal 570	red
	Rabbit monoclonal CD8	Abcam	ab217344	1/200	Opal 480	pink
Panel 3	Rabbit monoclonal FoxP3	Cell Signaling Technology	98388S	1/100	Opal 780	green
	Mouse monoclonal CD45RO	Cell Signaling Technology	14572S	1/1000	Opal 520	yellow
	Rabbit monoclonal GrazmB	Abcam	ab134933	1/200	Opal 620	brown
	Mouse monoclonal CK	Cell Signaling Technology	4545T	1/500	Opal 690	cycan

AJCC, American Joint Committee on Cancer; TC, tumor cell; IC, immune cell.

Supplementary Table 2. Association between TIGIT on TCs, TILs, and CD8+ T cells and clinicopathological parameters.

Variables	N	TIGIT on TCs			TIGIT on TILs			TIGIT on CD8+ T cells		
		Negative	Positive	P value	Negative	Positive	*P* value	Negative	Positive	*P* value
Sex				0.256			0.462			0.168
Female	120	17 (14.2)	103 (85.8)		8 (6.7)	111 (93.3)		33 (27.0)	89 (73.0)	
Male	144	28 (19.4)	116 (80.6)		13 (9.2)	128 (90.8)		28 (19.9)	113 (80.1)	
Age, years				0.332			0.866			0.224
<60	117	17 (14.5)	100 (85.5)		9 (7.8)	107 (92.2)		23 (19.7)	94 (84.3)	
≧60	147	28 (19.0)	119 (81.0)		12 (8.3)	132 (91.7)		38 (26.0)	108 (74.0)	
Location				0.068			0.348			0.252
Head & neck	161	22 (13.7)	139 (86.3)		11 (6.8)	150 (92.0)		34 (20.9)	129 (79.1)	
Body & tail	103	23 (22.3)	80 (77.7)		8 (8.2)	89 (89.9)		27 (27.0)	73 (73.0)	
Lymphovascular invasion				0.089			0.171			0.466
Absent	164	33 (20.1)	131 (79.9)		16 (9.9)	146 (90.1)		40 (24.7)	122 (75.3)	
Present	100	12 (12.0)	88 (88.0)		5 (5.1)	93 (94.9)		15 (21.7)	54 (78.3)	
Perineural invasion				0.597			0.244			0.823
Absent	85	16 (18.8)	69 (81.2)		9 (11.0)	73 (89.0)		19 (22.4)	66 (77.6)	
Present	179	29 (16.2)	150 (83.8)		12 (6.7)	166 (93.3)		42 (23.6)	136 (76.4)	
Tumor differentiation				0.36			0.938			0.072
Moderately/well-differentiated	166	31 (18.7)	135 (81.3)		13 (8.0)	150 (92.0)		44 (26.8)	120 (73.2)	
Poorly differentiated	98	14 (14.3)	84 (85.7)		8 (8.2)	89 (91.8)		17 (17.2)	82 (82.8)	
Tumor stage				0.73			0.435			0.739
T1-2	194	34 (17.5)	160 (82.5)		14 (7.3)	178 (92.7)		46 (23.7)	148 (76.3)	
T3	70	11 (15.7)	59 (84.3)		7 (10.3)	61 (89.7)		15 (21.7)	54 (78.3)	
Node stage				0.897			0.589			**0.001**
N0	102	17 (16.7)	85 (83.3)		7 (6.9)	94 (93.1)		34 (34.0)	66 (66.0)	
N1-2	162	28 (17.3)	134 (82.7)		14 (8.8)	145 (91.2)		27 (16.6)	136 (83.4)	
Distant metastasis				0.63			0.734			0.944
M0	255	44 (17.3)	211 (82.7)		20 (8.0)	231 (92.0)		59 (23.2)	195 (76.8)	
M1	9	1 (11.1)	8 (88.9)		1 (1.1)	8 (88.9)		2 (22.2)	7 (77.8)	
AJCC stage				**< 0.001**			0.792			0.665
I-II	104	35 (33.7)	69 (66.3)		16 (7.8)	188 (92.2)		49 (23.8)	157 (76.2)	
III- IV	169	10 (5.9)	159 (94.1)		5 (8.9)	51 (91.1)		12 (21.1)	45 (78.9)	

Supplementary Table 3. Association between CD155 on TCs and macrophages and clinicopathological parameters.

Variables	N	CD155 on TCs			CD155 on CD68+ macrophages		
		Negative	Positive	P value	Negative	Positive	P value
Sex				0.977			0.36
Female	120	25 (20.8)	95 (79.2)		49 (41.2)	70 (58.8)	
Male	143	30 (21.0)	113 (79.0)		51 (35.7)	92 (64.3)	
Age, years				0.262			0.93
<60	118	21 (17.8)	97 (82.2)		45 (38.5)	72 (61.5)	
≧60	145	34 (23.4)	111 (76.6)		55 (37.9)	90 (62.1)	
Location				0.406			**0.04**
Head & neck	161	31 (19.3)	130 (80.7)		54 (33.3)	108 (66.7)	
Body & tail	102	24 (23.5)	78 (76.5)		46 (46.0)	54 (54.0)	
Lymphovascular invasion				0.685			0.074
Absent	164	33 (20.1)	131 (79.9)		55 (34.0)	107 (66.0)	
Present	99	22 (22.2)	77 (77.8)		45 (45.0)	55 (55.0)	
Perineural invasion				0.221			0.068
Absent	85	14 (16.5)	71 (83.5)		25 (30.1)	58 (69.9)	
Present	178	41 (23.0)	137 (77.0)		75 (41.9)	104 (58.1)	
Tumor differentiation				0.874			0.527
Moderately/well-differentiated	165	34 (20.6)	131 (79.4)		65 (39.6)	99 (60.4)	
Poorly differentiated	98	21 (21.4)	77 (78.6)		35 (35.7)	63 (64.3)	
Tumor stage				0.112			0.211
T1-2	193	45 (23.3)	148 (76.7)		78 (40.4)	115 (59.6)	
T3	70	10 (14.3)	60 (85.7)		22 (31.9)	47 (68.1)	
Node stage				0.254			0.423
N0	102	25 (24.5)	77 (75.5)		42 (41.2)	60 (58.8)	
N1-2	161	30 (18.6)	131 (81.4)		58 (36.3)	102 (63.8)	
Distant metastasis				0.922			0.754
M0	254	53 (20.9)	201 (79.1)		97 (38.5)	155 (61.5)	
M1	9	2 (22.2)	7 (77.8)		3 (33.3)	6 (66.7)	
AJCC stage				0.149			0.143
I-II	206	47 (22.8)	159 (77.2)		83 (40.5)	122 (59.5)	
III- IV	57	8 (14.0)	49 (86.0)		17 (29.8)	40 (70.2)	

AJCC, American Joint Committee on Cancer; TC, tumor cell; IC, immune cell.
